# Subetta increases phosphorylation of insulin receptor β-subunit alone and in the presence of insulin

**DOI:** 10.1038/nutd.2015.20

**Published:** 2015-07-06

**Authors:** E A Gorbunov, J Nicoll, E V Kachaeva, S A Tarasov, O I Epstein

**Affiliations:** 1 OOO ‘NPF MATERIA MEDICA HOLDING', 47-1, Trifonovskaya street, Moscow 129272, Russian Federation; 2 Zen-Bio Inc., Research Triangle Park, NC, USA

## Abstract

It has been previously shown that Subetta (a drug containing released-active forms of antibodies to the insulin receptor β-subunit and antibodies to endothelial nitric oxide synthase) stimulated insulin-induced adiponectin production by mature human adipocytes in the absence of insulin. Therefore, it was assumed that Subetta could activate the insulin receptor. To confirm this hypothesis, the capacity of Subetta to activate the insulin receptor in mature human adipocytes in the absence or presence of the insulin was investigated. Cells were incubated either with Subetta or with vehicle, or with basal medium for 3 days. Then, adipocytes were treated with water or insulin (100 nm) for 15 min. Following treatment, lysates were prepared and phosphorylation of insulin receptor β-subunits was analyzed by western blot analysis. It was shown that Subetta significantly increased (*P*<0.001) the ‘phosphorylated-insulin receptor β-subunit/total insulin receptor β-subunit' ratios in both the presence and the absence of insulin. These results support previously published data and indicate that Subetta could activate the insulin receptor through the effect on its β-subunits, whose conformational state is essential for insulin receptor activation. This action might serve as one of the primary mechanisms of the drug's antidiabetic effect.

## Introduction

The starting point for the effects of insulin and related anabolic and catabolic processes is insulin binding to its receptor.^1^ Binding of insulin to extracellular α-subunits of the insulin receptor (IR) results in a conformational change that induces autophosphorylation of distinct tyrosine residues on the β-subunits leading to conformational changes in the intracellular β-subunits.^2, 3^

Various possible strategies for the research/design of compounds that act through the IR and its associated signaling pathways have been proposed previously:^2^ small ligands that mimic the natural ligand or stabilize a receptor–dimer interface, allosteric activators of IR, direct activators of the tyrosine kinase domain of IR or of the signal-transduction pathways, direct inhibitors of protein tyrosine phosphatase and so on. To date, numerous compounds, which use the proposed strategies, were developed and their antidiabetic efficacy was studied using both *in vitro* and *in vivo* models.^1, 4^

Subetta is a drug containing released-active forms of antibodies to the IR β-subunit and antibodies to endothelial nitric oxide synthase. A distinctive feature of the released-active antibodies is their ability not to suppress but to modify the activity of the antigen against which they were raised.^5^ Previously, Subetta demonstrated the ability to stimulate adiponectin production by mature human adipocytes in the absence of insulin.^6^ As it is known that insulin stimulates adiponectin secretion,^7^ we hypothesized that Subetta activates the IR in the absence of insulin in the culture medium via direct interaction with the IR β-subunit of mature human adipocytes.

Therefore, the aim of this work was to investigate the ability of Subetta to activate the IR β-subunit in mature human adipocytes in the absence or presence of insulin.

## Materials and methods

Human preadipocytes obtained from five healthy donors (sex: female; age: 47.0±5.4 years) (lot SL0047) were provided by Zen-Bio Inc. (Research Triangle Park, NC, USA) as described previously.^6^ All human tissues were procured from consented donors undergoing elective surgery under Institutional Review Board-approved protocols. Preadipocyte medium (catalog number PM-1), adipocyte differentiation medium (catalog number DM-2), adipocyte maintenance medium (catalog number AM-1) and adipocyte basal medium (catalog number BM-1) were provided by Zen-Bio Inc. Lysis buffer (Tris, pH 7.4, NP-40, NaCl, protease and phosphatase inhibitors) was the product of Zen-Bio Inc. IR β-subunit primary antibody (catalog number 3025), phospho-IR-β primary antibody (catalog number 3026) and α-rabbit immunoglobulin G coupled to horseradish peroxidase secondary antibody (catalog number 7074) were purchased from Cell Signaling Technology (Danvers, MA, USA). NuPage (4–12%) Bis-Tris polyacrylamide gels (catalog number NP0323Box), MOPS buffer (3-(*N*-morpholino)propanesulfonic acid; catalog number NP0001), SeeBlue Molecular Weight Markers (catalog number LC5700), nitrocellulose membrane (catalog number LC2001), Transfer buffer (catalog number NP0006) and NuPage LDS sample buffer (catalog number NP0007) were purchased from Invitrogen (Carlsbad, CA, USA). SuperSignal West Pico Kit (catalog number 34080) and Micro BCA Protein Kit (catalog number 23235) were purchased from Pierce/ThermoFisher (Rockford, lL, USA). Human recombinant insulin (catalog number 11376497001) was purchased from Roche (Mannheim, Germany).

Subetta manufactured based on patented biotechnological platform,^8^ as described previously,^6^ was supplied by ООО ‘NPF ‘MATERIA MEDICA HOLDING' (Russian Federation), as well as purified water (vehicle). The samples were provided encoded. The experiment and statistical analysis were performed blindly.

The assay used *in vitro* differentiated primary human subcutaneous adipocytes at 2 weeks post differentiation as described previously.^6^ Before assaying all the samples, the maximal possible amount of the sample, which could be used without cytotoxicity, was determined by standard MTT (3-(4,5-dimethylthiazol-2-yl)-2,5 diphenyl tetrazolium bromide) assay.

To determine if the test compounds activate the IR β-subunit in mature human adipocytes in the absence or presence of insulin, cells were treated for 72 h with test compounds in serum-free basal medium. After 72 h, cells were treated with water or 100 nm insulin for 15 min. Triplicate treatments were performed. Following insulin or water treatment, the medium was aspirated and the cells were washed three times with 2 ml per well of cold phosphate-buffered saline on ice. After the last wash, 250 μl per well of cold lysis buffer was added to each well. The cells were scraped off the plate and the lysate was transferred to 1.5 ml eppendorf tube on ice. The lysate was centrifuged at 10 000 *g* for 10 min at 4 °C. The supernatant was transferred to fresh tubes and the protein concentration was determined using Pierce Micro BCA Kit (Pierce/ThermoFisher) according to the manufacturer's instructions. The cell extracts were normalized for protein concentration and denatured by adding 2 × LDS sample buffer. The samples were briefly vortexed and centrifuged for 30 s at 10 000 *g* and further denatured at 75 °C for 5 min, and then centrifuged for 30 s at 10 000 *g*. Western blot assay: denatured samples were loaded onto 4–12% NuPage Bis-Tris polyacrylamide gels. Proteins were resolved during electrophoresis at 200 V in 1 × MOPS buffer according to the manufacturer's instructions. Prestained molecular weight markers were run in parallel to monitor the progress of electrophoresis as well as the transfer (see below). Following electrophoresis, proteins were transferred to nitrocellulose filters using the NuPage Transfer system. The transfer was performed in 1 × Transfer buffer containing 10% methanol for 60 min at 35 V. At the end of the transfer, the filters were briefly (15 s) rinsed in MilliQ H_2_O (EMD Millipore Corporation, Billerica, MA, USA). Following the water rinse, the blots were blocked for 30 min at room temperature in Tris-buffered saline, pH 7.6, containing 0.1% Tween-20 (TBS-T) and 3% bovine serum albumin. The blots then were incubated with primary antibody (IR β-subunit primary antibody or phospho-IR β-subunit primary antibody) overnight at 4 °C with gentle agitation. The blots were washed three times for 5 min each in TBS-T and reblocked for 30 min room temperature in TBS-T 3% bovine serum albumin. After blocking, the blots were incubated with α-rabbit immunoglobulin G coupled to horseradish peroxidase secondary antibodies at 1:3000 for 60 min at room temperature. The blots were washed 3 × 5 min each in TBS-T. After washing, the blots were incubated with chemiluminescent substrate for 5 min according to the manufacturer's protocol. Multiple images were obtained at various times of exposure (5–300 s) using a Bio-Rad ChemiDoc station (Bio-Rad Laboratories, Inc., Hercules, CA, USA). The quantity (intensity) of individual bands was obtained according to Bio-Rad ChemiDoc instructions using an equal volume rectangle. Comparison between samples was performed by first determining the ratio of signals detected in phospho-protein to total protein within a treatment sample. These values were then used to determine the ratio between control and treated samples. The average of the replicate values was determined and plotted as Fold Control for each treatment.

The data are presented as mean value per group (M)±s.d. Bartlett's test was used to confirm equality of variances between the groups. One-way analysis of variance followed by Tukey's honest significant difference test was used for statistical analysis and *P*-values <0.05 were regarded as significant (STATISTICA 6.1 software, StatSoft, Inc., Tulsa, OK, USA).

## Results and discussion

Based on the results of the MTT assay, the maximal amount of test compound that could be used without cell death was determined to be 1/2 maximal (1 part of test sample and 1 part of adipocyte maintenance medium) and this concentration was used for treatment (Table 1).

**Figure 1:**
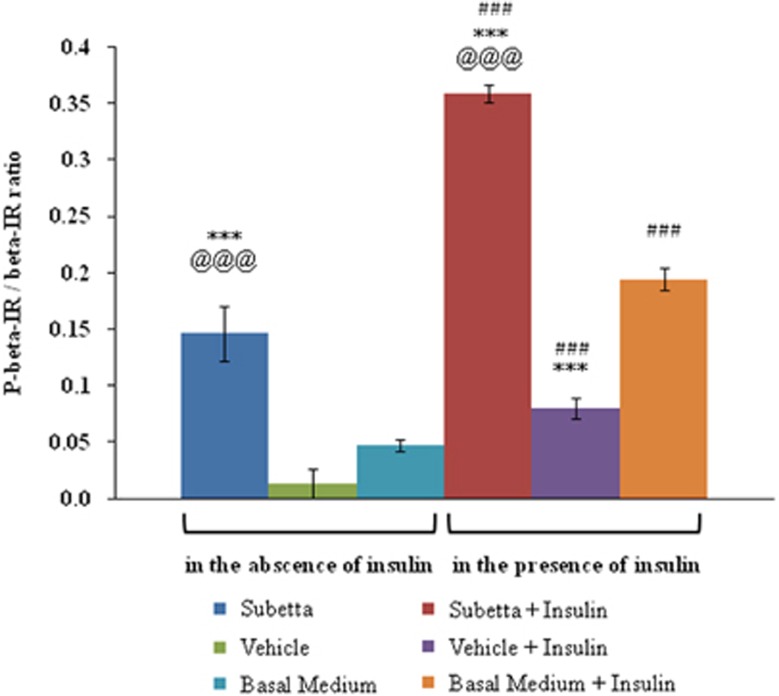
Effect of Subetta on ‘phosphorylated-IR β-subunit/total IR β-subunit' ratio. Data are expressed as means±s.d. from triplicate. One-way analysis of variance (ANOVA) followed by Tukey's honest significant difference test was used for statistical analysis. ANOVA shows the following results: F11/24=162.83, *P*=0.0000, observed power=1.0. Bartlett's test confirmed equality of variances between the groups (Bartlett test of homogeneity of variances data: result by group Bartlett's *K*^2^=10.3198, d.f.=11, *P*-value=0.5019, *P*>0.05). ***P<0.001 versus ‘basal medium' in the absence of insulin or versus ‘basal medium+insulin' in the presence of insulin; ^@@@^*P*<0.001 versus ‘vehicle' in the absence of insulin or versus ‘vehicle+insulin' in the presence of insulin; ^###^*P*<0.001 versus respective in the absence of insulin.

The incubation of mature human adipocytes with Subetta for 72 h followed by adding water or 100 nm insulin resulted in a statistically significant increase in ‘phosphorylated-IR β-subunit/total IR β-subunit' ratio (Figure 1). Subetta not only raised the ‘phosphorylated-IR β-subunit/total IR β-subunit' ratio by itself but also caused a greater increase in the ratio in combination with 100 nm insulin in comparison with insulin alone. The purpose of the study was not assessing kinetics of IR phosphorylation in the presence of the drug, and therefore one time point of insulin stimulation was used.

Vehicle did exert significant effect by itself but in combination with 100 nm insulin vehicle decreased the parameter. It is known that changing of ionic strength of the solution alters the protein–protein interaction by effect on the electrostatic contacts.^9, 10^ This negative impact following addition of the vehicle could explain why the phosphorylation of the IR was deteriorated. That is why the vehicle was used as control for Subetta—exactly the same vehicle was used in the manufacturing process of the drug.

This study is a direct and logical continuation of the first work,^6^ where the same experimental model was used to demonstrate the biological activity of Subetta *in vitro*. Here, it is shown that Subetta increases phosphorylation of the IR β-subunit by itself and enhances insulin's ability to stimulate phosphorylation of the protein. It is important to mention that Subetta's effect on the IR is more robust in the presence of insulin. This observation might be explained by double action on the IR: on the one hand insulin acts through IR α-subunits and on the other hand Subetta acts on IR β-subunits.

Our data suggest that Subetta could activate the IR through the effect on its β-subunits, whose conformational state is essential for IR activation. This action might serve as one of the primary mechanisms of the drug's antidiabetic effect.^11, 12^

## Figures and Tables

**Table 1:** Effects of vehicle on differentiated primary human subcutaneous adipocytes

*Sample*	*Sample amount*	*Optic density (M±s.d.) (*n*=6)*
Vehicle	Maximal (3.0 ml)	0.098±0.008
	1/2 Maximal (1.5 ml)	1.248±0.086
	1/3 Maximal (1.0 ml)	1.282±0.212
	1/4 Maximal (0.75 ml)	1.278±0.051
	0 ml	1.447±0.059

Abbreviations: M, mean; MTT, 3-(4,5-dimethylthiazol-2-yl)-2,5 diphenyl tetrazolium bromide.

At 2 weeks postdifferentiation as measured by the MTT assay.

## Notes

This work was funded by (OOO ‘NPF ‘MATERIA MEDICA HOLDING', Russian Federation). EAG, SAT, EVK and OIE have an affiliation to the commercial funders of this research study (OOO ‘NPF ‘MATERIA MEDICA HOLDING', Russian Federation) and JN is employed by a commercial company (Zen-Bio Inc., USA).
